# Validating Flow Cytometry as a Method for Quantifying *Bdellovibrio* Predatory Bacteria and Its Prey for Microbial Ecology

**DOI:** 10.1128/spectrum.01033-21

**Published:** 2022-02-23

**Authors:** Ayo Ogundero, Marta Vignola, Stephanie Connelly, William T. Sloan

**Affiliations:** a Infrastructure and Environment, School of Engineering, University of Glasgow, Glasgow, UK; Georgia Institute of Technology

**Keywords:** flow cytometry method (FCM), predatory bacteria, *Bdellovibrio*, quantitative polymerase chain reaction (qPCR), plaque forming unit, predator dynamics, cell enumeration

## Abstract

Bdellovibrio bacteriovorus is a predatory, Gram-negative bacteria that feeds on many pathogenic bacteria and has been investigated as a possible solution for mitigating biofilms in different fields. The application depends on more fundamental ecological studies into the dynamics between *Bdellovibrio* and their prey. To do so requires an accurate, reliable, and, preferably rapid, way of enumerating the cells. Flow cytometry (FCM) is potentially a rapid, accurate, and inexpensive tool for this, but it has yet to be validated in the enumeration of *Bdellovibrio*. In this study, we developed a protocol to measure the number of *Bdellovibrio* in samples of various densities using FCM and compared the results with those of other methods: optical density (OD), PFU assay (PFU), and quantitative PCR (qPCR). We observed a strong correlation between values obtained using FCM and PFU (ρ = 0.923) and FCM and qPCR (ρ = 0.987). Compared to optical density there was a much weaker correlation (ρ = 0.784), which was to be expected given the well-documented uncertainty in converting optical density (OD) to cell numbers. The FCM protocol was further validated by demonstrating its ability to distinguish and count mixed populations of *Bdellovibrio* and the prey Pseudomonas. Thus, the accuracy of FCM as well as its speed and reproducibility make it a suitable alternative for measuring *Bdellovibrio* cell numbers, especially where many samples are required to capture the dynamics of predator-prey interactions.

**IMPORTANCE** The rise of antibiotic resistance and the unwanted growth of bacteria is a universally growing problem. Predatory bacteria can be used as a biological alternative to antibiotics because they grow by feeding on other bacteria. To apply this effectively requires further study and a deeper understanding of the forces that drive a prey population to elimination. Initially, such studies require more reliable methods to count these cells. Flow cytometry (FCM) is potentially a rapid, accurate, and inexpensive tool for this, but it has yet to be validated for predatory bacteria. This study develops a protocol to count the predatory bacteria Bdellovibrio bacteriovorus and its Pseudomonas prey using FCM and compare the results with those of other methods, demonstrating its ability for studies into B. bacteriovorus predation dynamics. This could lead to the use of B. bacteriovorus for killing bacterial biofilms in fields, such as drinking water and agriculture.

## INTRODUCTION

Host-dependent strains of *Bdellovibrio* are a group of obligate predatory bacteria that kill and consume other bacteria to survive and reproduce (1). It is this ability to lyse cells as a function of reproduction that has led to increasing interest in *Bdellovibrio* as a biocontrol agent for their mitigation of biofilms (2). For example, the human gut and intestinal microbiota population are negatively affected by the excessive biofilm growth of Gram-negative bacteria but *Bdellovibrio* has been investigated as a potential probiotic to restore balance to the ecosystem and help treat conditions, such as inflammatory bowel diseases (IBD) (3).

Biofilms are described as the most common natural state of bacteria, where free-swimming planktonic bacterial cells group together and are embedded in a self-produced extracellular polysaccharide matrix, which helps to further anchor the cells to a substrate and facilitate continuous growth in this sessile state (4). This state increases bacteria’s tolerance to stresses such as antibiotics and other antimicrobial agents. The mechanisms providing this defense are not well characterized and vary depending on many factors such that biofilm-based infections are both persistent and difficult to control (4).

Effective use of predatory bacteria as a control agent for biofilms (2) requires a deeper understanding of how they interact with each other and the ecological dynamics with other microorganisms (5). Presently, our ability to explain the key factors that may influence *Bdellovibrio* predator-prey dynamics, such as predator-prey density, resistance, and space, is limited by how we enumerate both the predators and their prey. Many of the current experimental methods are cumbersome and arguably incapable of doing this accurately (6). Flow cytometry has recently been demonstrated as an inexpensive and versatile method to accurately count total and intact microorganisms with the ability to distinguish populations based on cellular features, such as size and nucleic acid content (7, 8). This has been applied to mixed cultures from many different environments, including drinking water (9), soil and sediment (10), and activated sludge (7, 11). *Bdellovibrio* has been investigated in relevance to these environments (3, 12–14) but the use of flow cytometry to quantify the predatory bacteria has yet to be compared to other conventional methods.

Measuring optical density (OD) or cell turbidity with a spectrophotometer is a common method to study bacterial growth (15). When light passes through a microbial culture it is scattered. This scattering is recorded by a spectrophotometer and can be used as an indication of the biomass present (16). OD is widely used, largely because of the speed and ease of measurement. This has also made the method a common preference for culture inoculation and harvest (17).

The small size of *Bdellovibrio* makes it difficult to directly measure the bacteria (18) so optical density is instead often used to indicate the suppression of a prey population in the presence of growing *Bdellovibrio* (19). A limitation here is that a decrease in optical density is shown with prey cell death regardless of cause, as such it provides only an indication of predation rather than a direct measure (6). Additionally, optical density is not as sensitive or accurate as other methods, making its use limited when trying to get a deeper understanding of predator dynamics (6).

The plaque forming unit (PFU) assay, also known as the double agar layer assay, is another of the most common laboratory techniques associated with *Bdellovibrio* (20). Here, dilutions of predatory bacteria prey are spread upon a cloudy lawn of prey bacteria on an agar plate to form clear zones in the agar. These clearings are known as plaques and are assumed to be formed by the initial growth of a single predator and are thus termed a plaque forming unit (21).

Like CFU plating techniques, the PFU assay is a popular and standard technique for obtaining a viable count because it is relatively accurate and requires less specialist equipment and reagents to perform than newer techniques, such as quantitative PCR (qPCR) (22). However, there is now an emphasis on the importance of high-throughput research in microbiology, which has called for new approaches because the PFU assay is time-consuming, slow, and vulnerable to human error (22).

In recent years genetic techniques have been widely deployed to accurately detect and quantify microbial populations (23, 24). qPCR is one method that has been increasingly used and seen as a high standard measure due to its speed, high sensitivity, and reproducibility (25).

qPCR offers accurate detection using several different approaches (26). To quantify *Bdellovibrio* populations in aquaculture zero discharge systems, one study made use of a *Taq hydrolysis* probe, a short fluorescent DNA sequence that is designed to complement and bind to a highly conserved region of the 16S rRNA specific to *Bdellovibrio* aquaculture (27). Using PCR, the targeted region is replicated in turn increasing the fluorescence produced. This fluorescence is recorded in real-time and used to accurately quantify, in absolute amounts, the initial number of target molecules (16S rRNA) and thus the organisms that carry them in the sample (28).

qPCR is mostly used to quantify either the total bacteria or a singular species present in environmental samples because it requires a large number of expensive reagents compared to flow cytometry, which makes it less suited in regular experimental work and in measuring the growth of samples with multiple species (29).

Flow cytometry (FCM) is a useful tool for quickly and reliably counting total microorganisms that have been stained with a fluorescence tag (usually SYBR green I) and enumerates cells via passage of the sample through a beam of laser light (9, 30) and, more advanced machines can sort and collect these cells (31).

Flow cytometry is also able to assess metabolic activity (32) and viability (33, 34) by use of a gating system to allow the user to distinguish and analyze cell populations of different properties (30). The FCM gating system has been used previously to distinguish bacteria populations in freshwater with low and high nucleic acid cell content based on their green fluorescence and side scatter measurements. Recently, advanced applications of FCM have further characterized the structure and phenotypic properties of subgroups within microbial communities to generate a unique fingerprint (35). As an alternative or in combination with molecular analysis it can establish the dynamics and biodiversity that contribute to the stability of microbial communities in natural and engineered systems (36–38).

In addition to the total cell count, flow cytometry can be used to estimate the number of nonviable cells by staining a population with propidium iodide (PI) and SYBR Green I (SYBR I) (39). PI can only enter bacteria with damaged cytoplasmic membranes, causing a reduction in the SYBR I to stain fluorescence, which allows those cells with intact membranes to be counted. This value is subtracted from the total count of the same sample to estimate the number of membrane-damaged cells (34). The ability of flow cytometry to distinguish between intact and membrane-damaged cells is important because *Bdellovibrio* can predate and reproduce in heat-killed cells with damaged membranes, which would alter any model of predator-prey dynamics (40).

Surprisingly, few studies are investigating *Bdellovibrio* predation using flow cytometry despite the size difference between the predator and their prey is reflected in the difference of forward-scattered (FSC) light signals in correlation with distinctive side-scattered (SSC) light signals, making it possible to distinguish *Bdellovibrio* from prey cells (41).

Direct cell count measurement using microscopy can also be considered a conventional method for measuring *Bdellovibrio* but is not included in this study. We believe PFU and qPCR, in particular, are sufficient as reliable and accurate measures of cell quantification to validate FCM. PFU is considered the standard method for determining *Bdellovibrio* viable count (18) and qPCR has also been shown to be the gold standard in measuring bacterial total count, especially when considering mixed cultures found in environmental samples (42, 43). Previous data have shown that direct cell counts and measurements by PFU are comparable for quantification of *Bdellovibrio* (44) but unlike PFU and qPCR, direct cell count measurements require a higher concentration per sample to measure accurately without the use of sophisticated hardware and software that may not be available in many labs (45). This makes the method less desirable and labor-intensive in many microbial ecology studies, such as growth time lapses.

This study aimed to determine whether flow cytometry could serve as a method for quantifying *Bdellovibrio* count as an alternative to the methods of optical density, the PFU assay, and qPCR. Filtered *Bdellovibrio* cell samples in a range of concentrations were prepared on different days and quantified by the four methods. The cell counts for flow cytometry were then compared to the other methods using a linear regression model and Pearson’s correlation coefficient (ρ). Additionally, the study aimed to show that flow cytometry could accurately count and distinguish mixed populations of *Bdellovibrio* and their prey Pseudomonas.

## RESULTS AND DISCUSSION

To validate flow cytometry as a suitable alternative to other methods of cell quantification, specifically optical density, the PFU assay, and qPCR, isolated samples of *Bdellovibrio* within a range of different concentrations (10^4^ to 10^9^ cells/mL) were quantified using each method. Linear regression and Pearson’s correlation analysis between the FCM results and the results obtained from each of the conventional methods were determined.

### Optical density.

**FIG 1:**
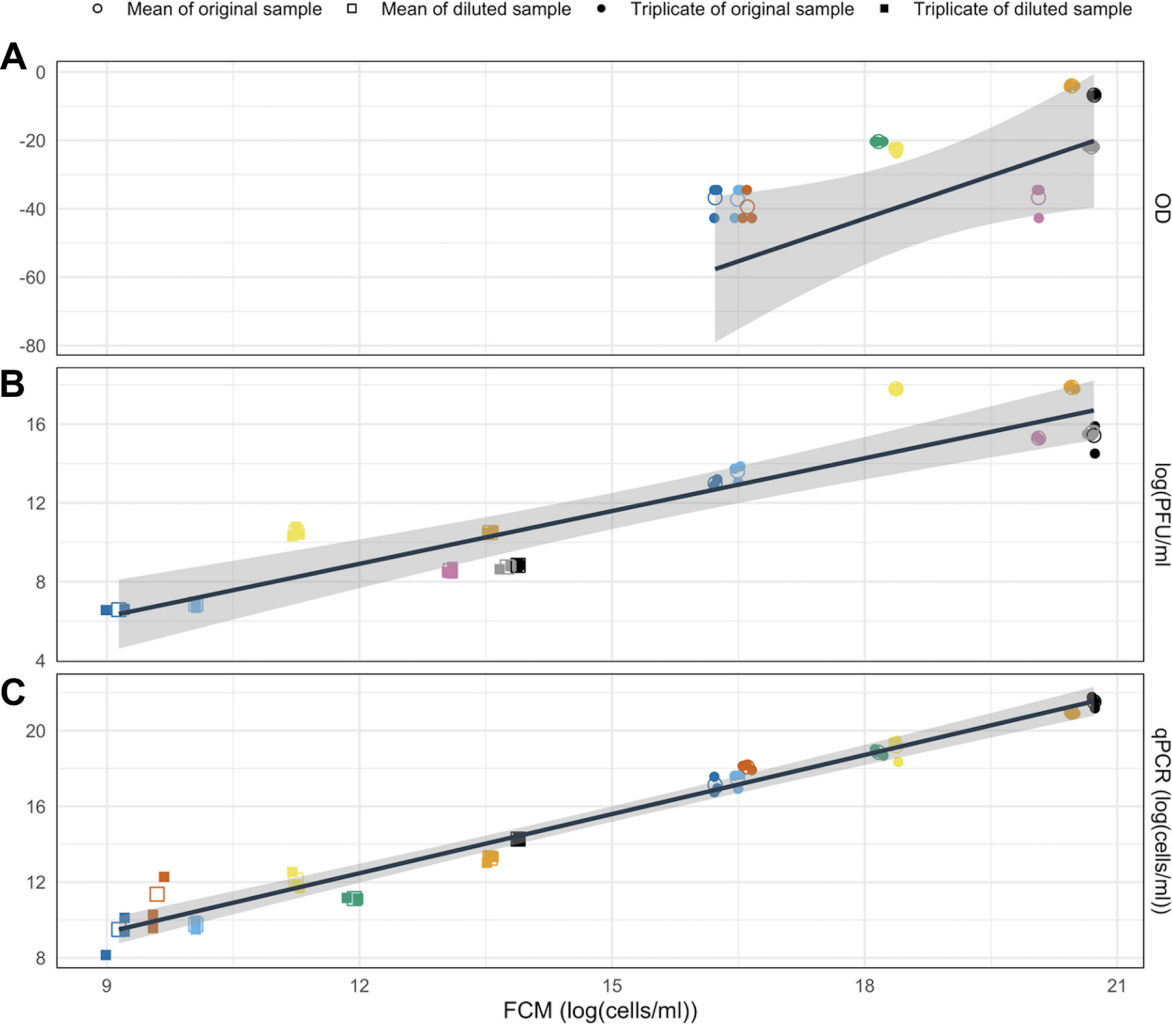
Linear regression plot with confidence intervals (gray) to compare quantification when using flow cytometry (log[cells/mL] with other methods): (A) Optical density (Box-Cox transformation, lambda = −0.6). Each bold point represents the mean of three observations. Pearson’s correlation coefficient: 0.784. *P* value < 0.05. Box-Cox transformed OD = −192.967 + logFCM*8.339. R^2^ = 0.456. (B) PFU method (log[PFU/mL]). Each bold point represents the mean of three observations. Pearson’s correlation coefficient: 0.923. *P* value < 0.0001. Log(PFU) = −1.821 + log(FCM^0.894^). R^2^ = 0.84. (C) qPCR (log(cells/mL) Each bold point represents the mean of three observations). Pearson’s correlation coefficient: 0.987. *P* value < 0.0001. Log(qPCR) = −0.023 + logFCM^1.041^. R^2^ = 0.972.

A linear relationship was observed between log-transformed measurements of cell count using FCM and the Box-Cox transformed measurements of optical density (Fig. 1A; correlation coefficient [ρ]: 0.784; *P* value < 0.05). However, this translates to a nonlinear relationship between the untransformed values. The low R^2^ value (0.456) suggested that enumeration provided by FCM cannot repeatedly and reliably predict the values provided by OD. This was expected and highlighted why optical density is not typically used to measure *Bdellovibrio* concentration.

The moderate correlation between the two methods could arise from differences in the material quantified and the means of detection of each measurement method. OD is not a direct measure of cell numbers, rather it is an indication of the total biomass concentration in a sample, including intact cells, damaged cells, and debris. Such debris, however, can contribute to the overall turbidity read by a spectrophotometer (46), thus resulting in an overestimation of cell count. In contrast, damaged cells and debris may be omitted from quantification using FCM by use of targeted gating and selective staining as applied here.

Further, the OD value only shows a linear correlation with biomass concentration at lower concentrations, the cutoff for which will differ depending on the spectrophotometer used and the path length of the cuvette used (16). The small size of *Bdellovibrio* also contributes to how difficult it is to accurately measure cell concentration (18). Quantification using OD provides a near-instant estimation of cell numbers with minimal sample preparation when measuring high concentrations, which is advantageous over the other methods applied here. One observation from this study was that the speed of OD as a measurement method and its moderate correlation between cell numbers as determined by FCM made optical density useful for estimating the required dilution necessary for a sample to be quantified by FCM and PFU assays.

### PFU assay.

A highly linear relationship was determined between the log-transformed relative cell numbers quantified by FCM and the PFU assay (Fig. 1B; correlation coefficient [ρ]: 0.923; *P* value < 0.0001), and the high R^2^ value (0.84) suggested that results provided by FCM were repeatable and reliable as an alternative to PFU for the assessment of relative cell numbers and that the relationship can be used to convert between measurements. Any increase in the cell numbers determined by FCM corresponds to a smaller increase in cells given by the PFU method (determined by the slope: 0.894). Differences in the count between the two methods are expected because the FCM protocol employed enumerates the total cells whereas the PFU method can only quantify the viable cells (47). Cells present in a sample that were dead or unable to replicate would not form plaques and, thus, would not be enumerated using the PFU method. In contrast, they would be enumerated using FCM. Nonviable cells may still be important to quantify when investigating *Bdellovibrio* because they could affect the living cell's ability to find prey when at high densities, which may play some role in maintaining predator and prey growth cycles. Additionally, were the method to be applied to the quantification of prey cells to map predator/prey dynamics, it has been shown that nonviable but intact Escherichia coli cells can still act as suitable hosts for *Bdellovibrio* growth (40).

Further, the discrepancy could occur because the cell count obtained from plating techniques often result in an underestimation because plaques can be formed by multiple cells originating close to each other, despite efforts to reduce this by sufficient diluting and spreading of the sample (47). Furthermore, while FCM may register false-positives, cells counted using PFU were less likely to be false-positives because plaques can only be achieved by the initial presence and sufficient replication of a lytic predator cell. In contrast, an observed count in flow cytometry was not necessarily specific to a *Bdellovibrio* predator cell and may be achieved in a number of different outcomes, such as bacterial cells of similar size or cell debris from prey cultures that may pass the filter during predator-prey separation. This study measured pure cultures of *Bdellovibrio* that were filtered from Pseudomonas, which are larger. Therefore, in this instance, flow cytometry was likely to show a high specificity. This also remains true for future studies involving predatory bacteria and Gram-negative host species, such as E. coli. Thus, FCM could allow the accurate enumeration of *Bdellovibrio* cells after long-term incubation with prey cells without the need of staining the cells before coculturing, which would limit the time of the study (48).

If enumeration of living cells were a priority for FCM instead of the total cell numbers as enumerated here, this could be achieved by using a live cell count assay, which makes use of both the SYBR I and PI dyes instead of a total cell count assay for FCM (49). It is anticipated that this could provide a better comparison to the PFU assay because this method can distinguish live and intact cells from those that have damaged membranes and would not be able to replicate and subsequently produce a plaque in the PFU method (50). Even so, it is important to note that even FCM estimates of live cell abundances are likely to be higher than the counts given by the PFU method because PFU methodology is more vulnerable to several biases, including human error, plaques originating from multiple cells that are close together or the influence of culture environment for the double layer agar plates which could result in the insufficient cultivation of bacteria (47).

### qPCR.

qPCR was used to measure the copy number of the *Bdellovibrio* 16SrRNA gene in a sample. This value was used to calculate the total cell count in a sample, based on reports of the copy number of the 16sRNA gene being two per single cell (51).

There was a significant correlation between the relative cell numbers quantified by FCM and qPCR (Fig. 1C) (correlation coefficient [ρ]: 0.987. *P* value < 0.0001, R^2^ 0.972), with qPCR proving to be a better-suited comparison to FCM than PFU. This is expected because unlike the PFU assay, qPCR and FCM are measures of the total cell number, so it is expected to be a better-suited comparison (25). The absolute cell numbers estimated by FCM have an almost perfect linear relationship with those given by the qPCR method (as determined by the slope: 1.041). However, the difference was negligible. As such, the data suggest FCM is an excellent method for quantification, enabling accurate and rapid quantification.

### Validation of FCM gating system.

**FIG 2:**
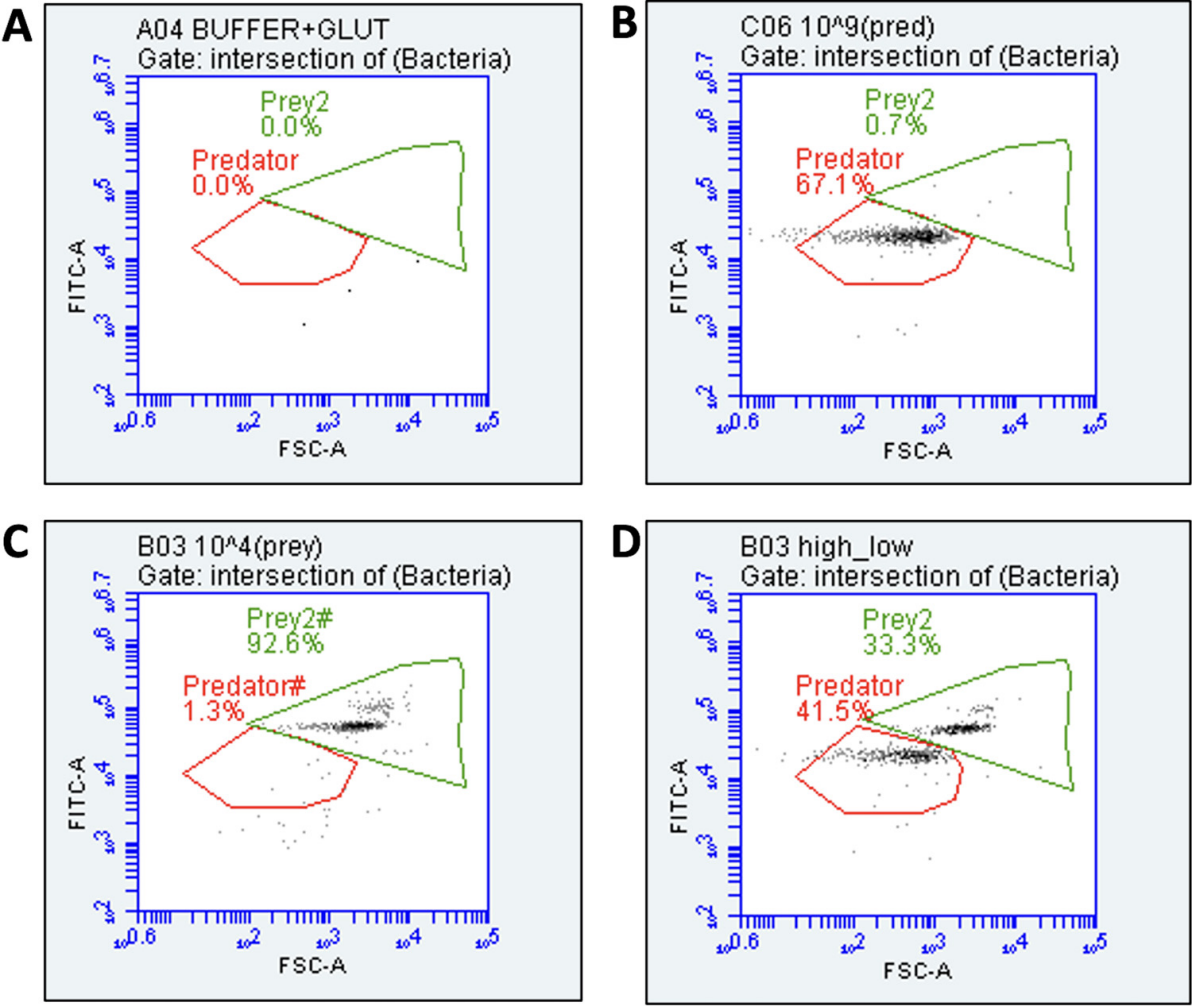
Dot plot of forward scatter (FSC-A) versus green fluorescence (FITC-A) of (A) growth buffer and glutaraldehyde (B) *Bdellovibrio* predator alone (C) Pseudomonas prey alone (D) Pseudomonas prey and B. bacteriovorus predator species mixed.

Whether measured alone or mixed with different concentrations of *Bdellovibrio*, the flow cytometry gating system (Fig. 2) was able to accurately quantify the Pseudomonas population.

**FIG 3:**
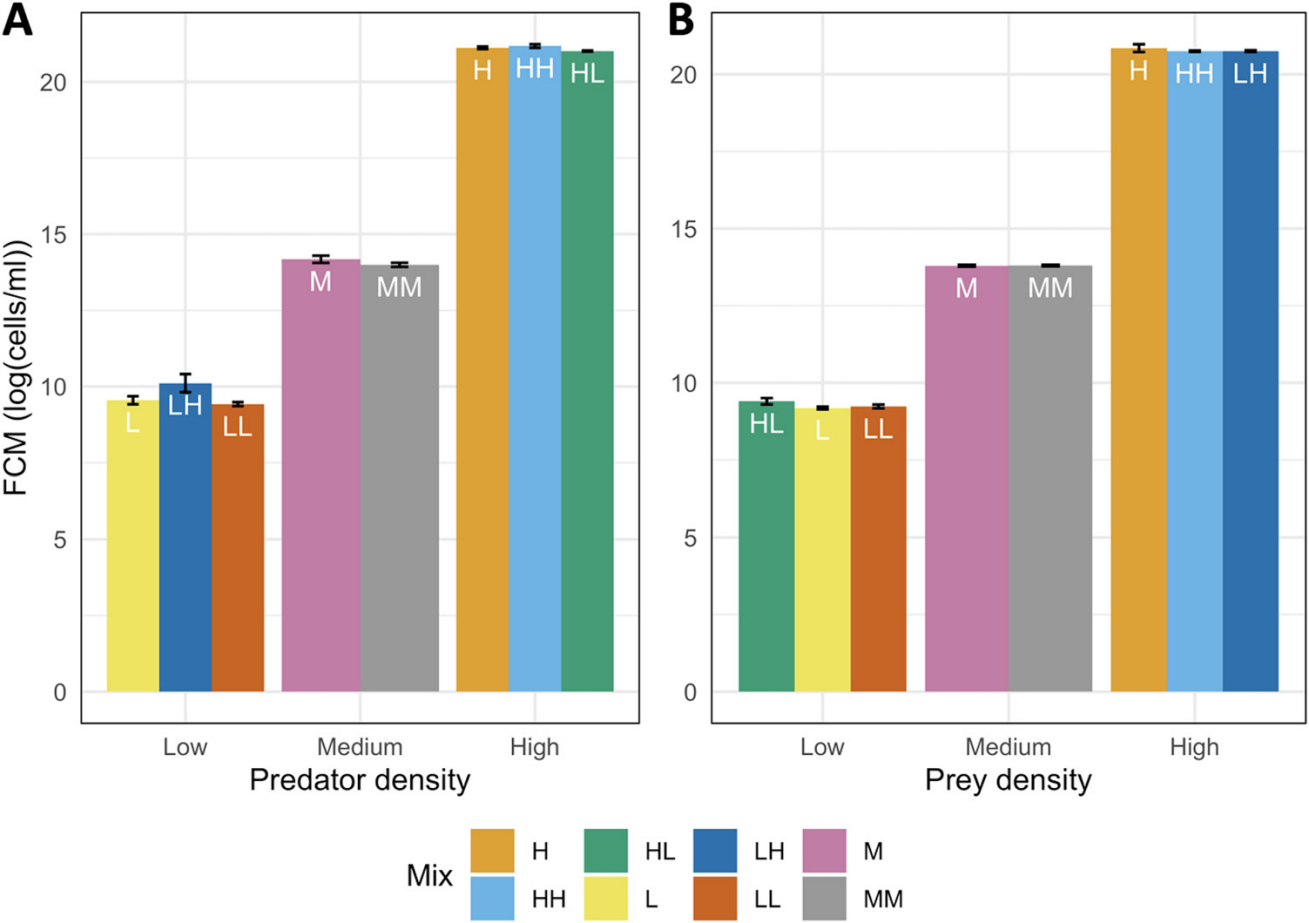
FCM count (log[cells/mL]) of the (A) *Bdellovibrio* predator and (B) the Pseudomonas prey at different densities (low, medium, and high) and different mix conditions: (L) monoculture of low density, (M) monoculture of medium density, (H) monoculture of high density, (LL) low predator: low prey densities, (LH) low predator: high prey densities, (HL) high predator: low prey densities, (HH) high predator: high prey densities and (MM) medium predator: medium prey densities.

As determined with the Kruskal Wallis test, no significant difference was found between FCM measurements of Pseudomonas samples of different mix ratios (Fig. 3; Kruskal Wallis test: *P* value > 0.05). Similar results were found with the *Bdellovibrio* samples, in that the FCM gating was able to accurately distinguish and count *Bdellovibrio* in lone or mixed samples. One discrepancy observed was that there was found to be a statistical difference between the predator samples of high concentration (Kruskal Wallis test: *P* value: 0.048). The Bonferroni test was applied to reveal that the difference in *Bdellovibrio* populations was specifically between the “high:high” versus the “high:low” predator: prey populations (*P* value: 0.048) whereas there was no significant difference in these samples when measured against the lone high predator sample (Bonferroni test, respective *P* values: 1 and 0.295). Although statistically different, both *Bdellovibrio* populations were measured to be the same order of magnitude (10^9^ cells/mL) and in practicality, the difference between the two is considered to be low, with the population in the “high: high” sample being a 20% increase from the “high: low” sample. Additionally, the choice of the threshold value for significance (*P* < 0.05) is largely subjective and a value of 0.01 could have also been chosen to make the test of significance more strict (52). Thus, with the other results, there is still confidence in FCM as a useful and accurate tool for quantifying species in a mixed culture using gating to identify each species, provided that the species are of a distinct size.

### General discussion.

FCM was demonstrated as an effective and rapid tool for enumerating both pure and predator-prey cocultures. Thus, it offers significant benefits over the conventional methods against which it was compared. Neither PFU nor qPCR can enumerate mixed cultures in a single assay. While OD can crudely enumerate total cell count in a mixed sample, OD measures can inaccurately enumerate cell concentration in a sample containing cells of varied size because larger cells can absorb and scatter more light (46). Thus, the application of optical density as a means of cell quantification is limited to monoculture samples because it cannot distinguish between different cells. Inaccurate results are produced when monitoring the growth of cultures in which multiple cell morphologies are exhibited, such as in a typical *Bdellovibrio*-prey coculture (53). Not only would the small size of *Bdellovibrio* contribute little to the differing light scattering properties of a sample that also contained much larger prey cells, such as Pseudomonas, but the included presence of the swollen Bdelloplasts would also increase the inaccuracy of the reading (22).

The direct application of FCM to the coculture of interest here was underpinned by the difference in the mean size of the two species in interest (B. bacteriovorus: 1.2 × 0.4 μm (54); Pseudomonas: 1 to 3 × 0.5 to 0.7 μm [55]), which, in turn, enabled a simple gating system to accurately quantify each species in a mixed sample. Gating systems like this cannot always be used alone even when investigating two populations of different sizes because the signal detected is a complex function of several parameters, such as particle orientation and cellular content (56). Thankfully, bacterial populations even in pure cultures display different levels of heterogeneity (57) and several options can detect this with advancement in flow cytometry (58, 59).

Flow cytometry has been shown to distinguish predatory bacteria and their prey by using fluorescent protein-expressing cells as an alternative to dyes for quantification was demonstrated by a previous study that incubated fluorescent tdTomato-expressing *B. bacterivorous* with different mutants of GFP-expressing Vibrio cholerae to investigate predator attachment (60). Distinguishing and quantifying the predator and prey in this could prove more specific as the gating strategy can easier pick out several differing fluorescents than when the gating strategy is determined largely by the forward scatter as is done in the current study. However, staining the cells after incubation in this manner would prevent the influence of long-term incubation on the photostability of the fluorescent protein, which could reduce the accuracy of quantification (61).

Another alternative would be to quantify bacteria using a 16SrRNA targeting fluorescent probe to combine flow cytometry with fluorescence *in situ* hybridization (FISH) (62). Although, there are no current studies that have used flow-FISH to quantify *Bdellovibrio* despite being a technique that has long been used to quantify several different bacteria in mixed populations (63). Additionally, FISH has previously been successful in the identification of *Bdellovibrio* cultured on E. coli and in environmental samples following enrichment (64). In flow-FISH, the probe used to stain the samples is similar to that used in qPCR, making it a more specific and sensitive method of quantification when using environmental samples that contain several different bacteria and typically have lower concentrations of *Bdellovibrio* (22).

In the case reported here, however, FCM’s ability to distinguish and count mixed predator: prey populations at the same time makes it ideal to study the effect of predator: prey density on predation. This represents a key factor for the application of *Bdellovibrio* as a biocontrol and is one that is yet to be investigated using flow cytometry. Several past studies have aimed to characterize the effect of predator: prey density; however, they have all used conventional techniques that may be limited (65). In addition, there remains debate over whether predation is favored in high or low predator: prey densities (66). Investigating this using new online flow cytometry technology, which uses automation, could be more accurate and easier to perform high-throughput and allow monitoring of growth continuously, for a longer duration (days to weeks), and in more detail so that a more complex model of predator-prey dynamics could be built (67, 68).

In conclusion, the tests performed show that flow cytometry represents a rapid and accurate alternative method for distinguishing and measuring *Bdellovibrio* and Pseudomonas cell counts compared to the methods of optical density, the PFU assay, and qPCR. Compared to optical density, a fast but largely inaccurate method, flow cytometry shows a nonlinear relationship. Compared to plaque forming unit, which is the most common method for *Bdellovibrio* enumeration, flow cytometry shows a nonlinear relationship with the raw variables but a strong linear relationship when both variables are log-transformed. In this case, the cell number measured using FCM overestimates those in plaque forming units, which is to be expected as PFU/mL, is a viable cell count only. Compared to qPCR, which is viewed as the gold standard for measuring 16srRNA as a proxy for cell count, FCM shows a very strong linear relationship. The population measured using FCM slightly underestimate those from qPCR, which was expected. Free DNA not present in cells can be detected by qPCR but not by the FCM gating system used. Because it has been validated as a suitable method, FCM can be used in further studies to measure *Bdellovibrio*, specifically in investigating the effect of density on predation and prey survival. Through this, further predator/prey population models can be constructed to aid their application in biofilm control.

## MATERIALS AND METHODS

### Bacterial strains and growth conditions.

The Bdellovibrio bacteriovorus strain HD100 (DSM no. 50701) was used throughout this study and was grown by predation on Pseudomonas putida. (DSM no. 50906) using standard culturing methods (69, 70). Pseudomonas cells were grown in LB broth at 30°C with shaking (150 rpm) for 16 h and resuspended in supplemented Ca/Mg-HEPES buffer (25 mM HEPES, 2 mM calcium chloride, 3 mM magnesium chloride pH 7.6) to an optical density (600 nm) value of 10. This prey suspension was stored at 4C for later use. Fifty microliters of B. bacteriovorus from a glycerol stock stored at −80°C was added to 1 mL of the prey suspension in 10 mL dilute nutrient broth at 30°C with shaking (200 rpm) for 24 h and then subcultured twice at 24 h intervals by transferring 200 µL of the culture and 1 mL of the prey suspension to 10 mL Ca/Mg-HEPES buffer (predator-prey ratio 1:10). In total seven replicate cultures were prepared for use in the quantification of B. bacteriovorus in pure culture. For the validation of the FCM gating system, an eighth culture was also grown as described. Additionally, a pure Pseudomonas culture was grown in LB for 16 h.

### Preparation of predator filtrate samples for enumeration and comparison.

Seven replicate cultures were grown as described then prepared for enumeration. To remove the prey and harvest the predator alone each culture was filtered twice through a 0.45 µm pore size syringe filter (Fisherbrand). An aliquot of each filtrate was then diluted by 10^−3^ times to produce two *Bdellovibrio* samples from each culture; the original at a ‘high’ concentration (∼10^7^ to 10^9^ cells/mL) and the dilution of ‘low’ concentration (∼10^4^ to 10^6^ cells/mL). Through this, the concentration range of the prepared samples was estimated to be from 10^4^ to 10^9^ cells/mL. The average cell counts of predator filtrate samples were then obtained from triplicate measurements using flow cytometry and compared with three quantification methods: optical density (OD), qPCR, and the plaque forming unit (PFU) assay. An aliquot of each filtrate is plated on a solid LB agar plate to confirm the removal of prey.

### Flow cytometry.

Total cell count measurements of each of the high and low concentration predator filtrate samples were performed using a Bd Accuri C6 plus flow cytometer. Samples were initially fixed 1:1 vol/vol with glutaraldehyde (1% in DI water) stored in the dark at 4°C and analyzed within 1 h. Before staining, where necessary, samples were diluted to achieve an events per second reading of less than 600 on the flow cytometer. Dilutions were made in filtered (0.22 µm Sartorius Minisart Plus Syringe Filters, Fisher scientific) DI water. The samples were each stained with 10 µL/mL of SYBR green I (10,000× in DMSO, Thermofisher) previously diluted 1:100 in Ethylenediaminetetraacetic acid (1 mM, Sigma-Aldrich) and incubated in the dark at 37°C for 13 min before measurement (9).

Gating was used to distinguish selected signals (B. bacteriovorus and Pseudomonas cells) from each other and the background (inorganic and organic particles) using a dot plot of forward scatter (FSC-A) versus green fluorescence (FITC-A). This was achieved with the aid of negative controls consisting of the deionized water used for dilutions, HEPES buffer used for growth, and a sample of the predator/prey coculture further filtered (0.22 µm) to remove any bacterial cells.

### Optical density.

To measure the OD of each of the high and low concentration predator filtrate samples, 1 mL of each sample was aliquoted in polystyrene semi-micro cuvettes (Fisherbrand) for measurement and each sample was measured in triplicate by optical density at 600 nm (OD_600_) using the Hach DR 2800 Portable Spectrophotometer. Due to the lack of sensitivity with the spectrophotometer, optical density was only used to measure samples from 10^7^ to 10^9^ cells/mL as quantified by flow cytometry.

### PFU assay.

The plates used for the PFU assay comprised two layers. The bottom layers (dilute nutrient broth, 1.5% [wt/vol] agar, supplemented with 2 mM calcium chloride and 3 mM magnesium chloride) of the double layer agar plates were prepared in advance. To plate, an aliquot of each of the high and low concentration predator filtrate samples was prepared in a range of 10-fold dilutions. Triplicates of each dilution were then mixed carefully with 500 µL of a Pseudomonas suspension (OD = 10) and dilute nutrient broth, 0.7% (wt/vol) agar supplemented with 2 mM calcium chloride and 3 mM magnesium chloride. This mixture was then poured over the base of the double-layer agar to form the top layer. Once dry, the plates were incubated at 30°C for 3 to 4 days and the number of formed plaques were counted. The PFU/mL was read from a plate with 30 to 300 plaques, the original sample concentration was calculated from the volume plated, and the dilution used as in the equation PFU/ml = number of plaques/(dilution factor × volume (ml) plated).

### Standard curve preparation for qPCR.

The qPCR was performed for each of the high and low concentration predator filtrate samples of B. bacteriovorus to enumerate the total copy number of the 16S rRNA gene (492 bp conserved locus-specific to the *Bdellovibrionaceae* family) based on a previously developed protocol (51).

The total 16S rRNA gene copy number of a sample was determined by comparison with a standard curve of known concentrations of a plasmid containing the 16S rRNA gene. The number of cells was inferred from the value of the total copy number based on reports of *Bdellovibrio* having an approximate copy number of 2 16S rRNA genes per cell (71).

To amplify a fragment of 492 bp of the B. bacteriovorus HD100 16S rRNA gene to use as a standard for qPCR, PCR was performed on pure cultures using a protocol previously developed (51). The PCR contained 12.5 μL of PCR master mix (Lambda Biotech), 1 μL of each primer (10 μm; BbSF216: 5′‐TTTCGCTCTAAGATGAGTCCGCGT‐3′ and BbSF707: 5′‐TTCGCCTCCGGTATTCCTGTTGAT‐3′) previously designed (72), 2 μL of DNA, and 8.5 μL of PCR grade water. Positive (*Bdellovibrio* DNA) and negative (no DNA) controls were included.

The PCR was performed with a GeneTouch thermal cycler (Bioer) using a thermal profile of denaturation and enzyme activation at 95°C, 2 min, followed by 36 cycles of 95°C for 30 s, annealing at 51°C for 30 s, extension at 72°C for 30 s, and final extension at 72°C for 5 min. PCR products were verified by gel electrophoresis (1% agarose gel in 0.5%) Tris acetate-EDTA (TAE) buffer stained with 10 μL of SYBR Safe DNA gel stain (Thermofisher) at 110 V for 1 h, and the gel was digitized with a Gel Doc XR+ imager (Bio-Rad).

The standard fragment was then cloned with the pGEM-T easy plasmid vector system (Promega). The DNA was quantified using a NanoDrop ND-1000 UV-Vis Spectrophotometer (Thermofisher) and a 10-fold dilution series for the standard curve was prepared from 9 to 9 × 10^6^ plasmid copies per reaction using a calculation previously described (51). qPCR for the standard curve was always performed in combination with the qPCR assays for the *Bdellovibrio* samples.

### qPCR.

After preparation of the high- and low-concentration predator filtrate samples, aliquots of each were first frozen at −80°C to ensure complete lysis of the cells. As in Van Essche et al., the qPCR assay used primers Bd347F (5′-GGAGGCAGCAGTAGGGAATA-3′) and Bd549R (5′-GCTAGGATCCCTCGTCTTACC-3′) and a TaqMan nucleic acid staining probe, Bd396P (5′-TTCATCACTCACGCGGCGTC-3′), which is labeled with 5′ FAM (6-carboxyfluorescein) and 3′TAMRA (6-carboxytetramethylrhodamine) (51). The thermal profile was also as stated previously with the data collected during the annealing and extension steps (51). Reactions were run in a C1000 thermal cycler (Bio-Rad) and included 12.5 μL of Jumpstart *Taq* Readymix for High Throughput Quantitative PCR (Sigma-Aldrich), 2 μL of each primer (10 μM), 1.25 μL of the TaqMan probe (1 μM), 5 μL of DNA and 2.25 μL PCR grade water as previously described (27). The reaction efficiency was calculated using the slope of the standard curve to be on average 104.34% with an R^2^ value of 0.99.

### Validation of FCM gating system.

To confirm that the FCM gating system developed was accurate in distinguishing between *Bdellovibrio* and their larger prey, Pseudomonas, a sample each of pure *Bdellovibrio* filtrate and Pseudomonas were prepared separately to ∼10^9^ cells/mL as above. Aliquots of each sample were prepared and then diluted from high (10^8^ to 10^9^ cells/mL) to medium (10^6^ to 10^7^ cells/mL) and low (10^4^ to 10^5^ cells/mL) concentrations and measured in triplicate using FCM as described above.

Following this, aliquots of the prey and predator with known concentrations were mixed in different ratios and again measured in triplicate using FCM. The preparations were as follows: high predator: high prey (HH), high predator: low prey (HL), medium predator: medium prey (MM), low predator: high prey (LH), and low predator: low prey (LL).

### Statistical analysis.

The statistical analysis was performed using R software. Pearson’s correlation coefficient (ρ) was calculated to find the relationship between cell quantification by FCM and the other methods. Linear regression models were used to find the significant differences in measurement between FCM and the other methods tested. Natural log transformations of FCM, PFU, and qPCR values were used while the OD values were transformed using the BoxCox method (Table S1). This was to allow the data to satisfy assumptions of normality and homoscedasticity. Note that when the gradient of the relationship between log-transformed variables is one it reflects a perfectly linear relationship between the raw, untransformed variables.

To validate the gating system, Kruskal Wallis (k-w) and Bonferroni tests were used to find the significant difference between sample preparations (mixed versus pure) of the same concentration.
