# Supplementary material for: Validating Flow Cytometry as a Method for Quantifying Bdellovibrio Predatory Bacteria and Its Prey for Microbial Ecology
Source: Microbiol Spectr. 2022 Feb 23;10(1):e01033-21. doi: 10.1128/spectrum.01033-21 (PMC8865432; doi:10.1128/spectrum.01033-21)
Supplement: SUPPLEMENTAL FILE 1 — Supplemental material. Download SPECTRUM01033-21_Supp_1_seq5.pdf, PDF file, 0.1 MB [file spectrum01033-21_supp_1_seq5.pdf]

<u>Table s1</u>- The mean and standard deviation for optical density (600nm) values from three observations and the Box-Cox transformations (lambda=-0.6).

OD(600nm)	Standard deviation	Box-Cox transformed OD
0.00233333	0.00057735	-61.637923
0.00266667	0.00057735	-57.607402
0.00266667	0.00057735	-56.801539
0.00266667	0.00057735	-56.713789
0.00666667	0.00057735	-32.227299
0.007	0	-31.051573
0.008	0	-28.532486
0.052	0.002	-8.156392
0.11	0.01	-4.599662
